# Chirality-Dependent Interaction of d- and l-Menthol with Biomembrane Models

**DOI:** 10.3390/membranes7040069

**Published:** 2017-12-15

**Authors:** Pooja Gusain, Shinya Ohki, Kunihide Hoshino, Yoshio Tsujino, Naofumi Shimokawa, Masahiro Takagi

**Affiliations:** 1 School of Materials Science, Japan Advanced Institute of Science and Technology, 1-1Asahidai, Nomi, Ishikawa 923-1292, Japan; s1440152@jaist.ac.jp (P.G.); shinya-o@jaist.ac.jp (S.O.); kunihide_hoshino@takasago.com (K.H.); ytsujino@jaist.ac.jp (Y.T.); nshimo@jaist.ac.jp (N.S.); 2 Takasago International Corporation, 5-37-1, Kamata, Ota-ku, Tokyo 144-8721, Japan

**Keywords:** d- and l-menthol, phase separation, lipid rafts, liquid-ordered (L_o_), liquid-disordered (L_d_), solid-ordered (S_o_)

## Abstract

Chirality plays a vital role in biological membranes and has a significant effect depending on the type and arrangement of the isomer. Menthol has two typical chiral forms, d- and l-, which exhibit different behaviours. l-Menthol is known for its physiological effect on sensitivity (i.e., a cooling effect), whereas d-menthol causes skin irritation. Menthol molecules may affect not only the thermoreceptors on biomembranes, but also the membrane itself. Membrane heterogeneity (lipid rafts, phase separation) depends on lipid packing and acyl chain ordering. Our interest is to elaborate the chirality dependence of d- and l-menthol on membrane heterogeneity. We revealed physical differences between the two optical isomers of menthol on membrane heterogeneity by studying model membranes using nuclear magnetic resonance and microscopic observation.

## 1. Introduction

At lower temperature, the phase separation between the liquid-ordered (L_o_) phase and liquid-disordered (L_d_) phase are spontaneously formed in multicomponent lipid membranes consisting of saturated lipid, unsaturated lipid, and cholesterol. In particular, the L_o_ phase composed of the large amounts of saturated lipid and cholesterol has attracted great attention in association with the “raft region”. Lipid rafts are specialized membrane microdomains enriched in saturated lipids, sphingolipids, and cholesterol found in the plasma membrane [1,2,3,4,5,6,7]. Moreover, lipid rafts are believed to serve as a platform for the regulation of various cellular processes involved in signal transduction and membrane trafficking [8,9]. The fatty acid chains of phospholipids present in the lipid raft regions are rather tightly packed in the L_o_ phase, unlike the L_d_ phase present in the bulk of the membranes. Therefore, many studies have investigated the phase separation between L_o_ and L_d_ phases as a simple model for the raft domain.

Some membrane proteins, such as ion channels, are localized on lipid rafts. The activity of a channel is controlled by specific molecules, and changes in activity resulting from molecular binding in a channel generates a sensation, such as warmth, cold, or anaesthesia [10]. This binding molecule may not only affect the channel, but also the lipid bilayer, thus altering the physical properties of the membrane. Therefore, channel activity is also influenced through the lipid membrane, and specific changes in the physical properties of lipid membranes by several sensing molecules have been revealed [11]. There are some important parameters regarding to the relative stability of the various packing geometries in the lipid bilayer, such as hydrophobic acyl chain ordering and hydrophilic head group interaction [12]. These arrangements of lipids are determined by the balance of interactions at the interface of the head group (steric repulsion and electrostatic interaction) and within the hydrophobic region (hydrophobic interaction and van der Waals attraction) [13]. Hence, it becomes very important to perceive these structural changes in lipids on their interaction with such sensing molecules.

The effects of external molecules on the physical properties of a membrane have been studied using giant unilamellar vesicles (GUVs). GUVs have attracted considerable attention as biomimetic model membranes of living cells because they mimic actual cell structures. GUVs one micrometre in diameter [14] are large enough to allow real-time observation by optical microscopy [15,16]. Since the lipid composition of liposomes can be easily changed, they are commonly used to investigate the effects of lipid components. A recent study suggested that the heterogeneous distribution of membrane lipids results in the formation of phase-separated membranes which mimic lipid rafts. Heterogeneous phase-separated structures in multicomponent systems can be easily observed as solid-ordered (S_o_), L_o_, and L_d_ domains [17,18,19]. 

Almost all organisms can detect ambient temperature through primary afferent sensory neurons over a wide range of temperatures from noxious cold to noxious heat. There are two ways to detect these temperatures, either through transient receptor proteins (TRP) channels present in sensory neurons or TRP channels in the skin. TRP channels are not only activated by the temperature, but also respond to a variety of chemical agonists. Menthol is an interesting channel-activating molecule because it plays an important role in sensation pathways. Menthol triggers a sensation of cold in the brain. Nerves carry information in the form of electric currents and thereby sense changes in temperature. The protein transient receptor potential cation channel subfamily M member 8 (TRPM8) senses changes in temperature and provides a cooling sensation upon the application of menthol. TRPM8 is also known as the cold and menthol receptor 1 (CMR1), and in humans is encoded by the TRPM8 gene [20]. TRPM8 is an ion channel. Activation of this ion channel allows the entry of Na^+^ and Ca^2+^ into the cell, leading to depolarization and the generation of an action potential. The entry of Ca^2+^ through ion channels is regarded as a second messenger of all the signalling processes ranging from cell differentiation to growth to cell death. The signal is conducted from primary afferents (type C- and A-delta), eventually leading to the sensation of cold and cold pain. Plasma membrane also locates voltage-dependent calcium channels, which are highly selective in nature and are responsible for opening of the transmembrane pore [21]. Gandini et al. highlighted the potential application of the peptide toxins in relation to structural functional activity of voltage-dependent calcium channels and their role in human diseases [22]. Menthol shows dual behaviour, depending on the temperature: it enhances cooling sensations at room temperature, whereas above 37 °C, it enhances the sensation of warmth [23,24,25]. l-Menthol is a versatile compound and interacts with many receptors such as TRPM8, transient receptor potential cation channel subfamily V member 3 (TRPV3), transient receptor potential cation channel subfamily V member 1 (TRPV1), and transient receptor potential cation channel subfamily A member 1 (TRPA1) [23]. Kappa opioid receptors are also stimulated by l-menthol and enhance the voltage-temperature-dependent gate mechanism. Furthermore, it is believed that prolonged exposure to l-menthol will cause the desensitization to cold sensitive fibres, similar to capsaicin causing the self-desensitization of heat-sensitive TRPV1 [26]. It was also reported that the C-terminus of TRPM8 channel contains structural elements, which are important in temperature-dependent gating. However, the mechanism behind those temperature-dependent changes is still controversial at the molecular level.

The complexity of living systems is still a greater challenge to understand the importance of chirality in various biological processes. Biological molecules are often chiral, such as DNA, proteins, carbohydrates, lipids, and steroids. Menthol is also chiral. There are several “diasteriomers” of menthol, of which the most common optical isomers are (1S, 2R, 5S)-2-isopropyl-5-methylcyclohexanol (d-menthol) and (1R, 2S, 5R)-2-isopropyl-5-methylcyclohexanol (l-menthol). l-Menthol is the main form found in nature and is widely used in products such as toothpaste, chewing gum, and cigarettes [27,28,29,30]. l-Menthol is believed to bind onto TRPM8 and causes a well-known, desired, cooling sensation [31,32]. In contrast, d-menthol does not exist in nature and does not induce a cooling sensation. Many quantitative analyses were performed about structure-function relationship of different optical isomers of menthol. All forms, i.e., pure (l- and d-menthol) and racemic (dl-menthol), exhibit different properties. Some studies revealed that menthol acts stereoselectively in biological systems. Hall et al. showed the stereoselectivity of l-menthol on GABA_A_ currents compared to d-menthol [33]. On the other hand, Abrar et al. were not able to detect a stereoselectivity of menthol actions on the α7-nACh receptor [34]. This difference in properties makes only l-menthol affect the cold receptor response, whereas the other does not. Similarly, studies on different chiral conformations attained by organocobalt complexes have been reported in the past. Self-organization of these flexible chiral organocobalt complexes is believed to proceed enantioselectively [35,36]. Hence, chirality plays a crucial role in the functioning of the molecules in biological processes. Although the importance of chirality for proteins has been discussed, the underlying significant differences in the interaction between d-/l-menthol and the lipid membrane are not well understood. The first step in addressing this is to elucidate how and where d-/l-menthol each interact with a membrane. 

In this paper, we used GUVs as model membranes (artificial cells) to examine the interaction between a lipid membrane and d-/l-menthol. Several techniques were used: nuclear magnetic resonance (NMR), fluorescence microscopy, and confocal laser scanning microscopy. First, we performed NMR experiments to identify the interaction sites of d- and l-menthol with model membranes. Second, we observed the effect of d- and l-menthol on phase-separated membranes composed of dioleoylphosphocholine (DOPC)/dipalmitoylphosphocholine (DPPC)/cholesterol (Chol) with varying concentrations of Chol (0%, 20%, and 30% (*v*/*v*)). Finally, we demonstrated that d- and l-menthol have different effects on a membrane, depending on the sterol concentration.

## 2. Materials and Methods

### 2.1. Materials

1,2-Dioleoyl-*sn*-glycero-3-phosphocholine (DOPC), 1,2-dipalmitoyl-*sn*-glycero-3-phosphocholine (DPPC), and cholesterol (Chol) were purchased from Avanti Polar Lipids (Alabaster, AL, USA). (1*S*, 2*R*, 5*S*)-2-Isopropyl-5-methylcyclohexanol (d-menthol) and (1*R*, 2*S*, 5*R*)-2-isopropyl-5-methylcyclohexanol (l-menthol) were purchased from Wako Japan. The fluorescent dye *N*-(rhodamine red-X)-1,2-dihexadecanoyl-*sn*-glycero-3-phosphoethanolamine triethylammonium salt (rhodamine DHPE) (λ_ex_ = 560 nm, λ_em_ = 580 nm) was obtained from Thermo Fisher Scientific (Waltham, MA, USA). Chloroform and methanol were from Kanto-Chemical (Japan) and Nacalai Tesque (Kyoto, Japan), respectively. Ultrapure water (specific resistance ≥ 18 MΩ) was obtained using a Millipore Milli-Q purification system.

### 2.2. NMR Measurements

All NMR measurements were carried out on an AVANCE III 800 spectrometer equipped with a TCI cryogenic probe, at a ^1^H frequency of 800.23 MHz (Bruker Biospin, Rheinstetten, Germany). Titration experiments of DOPC and Chol with d- or l-menthol were monitored by ^1^H- and ^13^C-NMR. All samples were dissolved in CDCl_3_, and 4, 4-dimethyl-4-silapentane-1-sulfonic acid (DSS) was used as a chemical shift reference. The examined compositions were DOPC, DOPC/Chol (1:0.05) and DOPC/d- or l-menthol (1:0.1). The sample temperature was maintained at 15 °C during the NMR experiments. Recorded FIDs were processed and analysed using the software package, TOPSPIN ver. 3.2. 

### 2.3. Preparation of Liposomes

Lipid vesicles were prepared by the natural swelling method [19]. Lipids, Chol, menthol, and Rho-DHPE fluorescent probes were dissolved in a 2:1 *v*/*v* (chloroform/methanol) solution, to give concentrations of 2 mM for lipids, Chol, and menthol, and 0.1 mM for Rho-DHPE. Lipids, Chol, and menthol were mixed at the desired composition to give a total volume of 20 μL. Rho-DHPE (2 μL) was further added. The organic solvent was evaporated under a flow of nitrogen gas and the lipids were further dried in a vacuum desiccator for 3 h. The film was hydrated with 200 µL Milli-Q water at 37 °C for one hour and then kept overnight at room temperature (21.7 ± 1.7 °C). The final lipid concentration was 0.2 mM and the Rho-DHPE concentration was 1 μM respectively. The prepared lipid compositions for microscopic observation were DOPC/DPPC/Chol = 50:50:0, 40:40:20, and 35:35:30 as control systems without menthol. When we add 10 mol % menthol to lipid compositions, we fixed DOPC:DPPC:Chol = 1:1:0, 2:2:1, and 7:7:6, that is DOPC/DPPC/Chol/d- or l-menthol = 45:45:0:10, 36:36:18:10, and 31.5:31.5:27:10.

### 2.4. Microscopic Observations

Lipid vesicle solution (5 µL) was placed in a silicone well (0.2 mm) on a glass slide and covered with a small cover slip. The silicone well and coverslip ensured that no evaporation of the solution occurred during the experiment. The formed GUVs were observed by fluorescence microscopy (Olympus IX71, Tokyo, Japan) at room temperature (22.8 ± 2 °C). The images were recorded on a hard disc drive at 30 frames/s. GUVs were prepared by using the unsaturated lipid DOPC, the saturated lipid DPPC, and Chol to form two phase membranes with lateral heterogeneity. The heterogeneous structure was observed by incorporating the fluorescent probe rhodamine-DHPE, which partitioned into the DOPC-rich region. To avoid the photo-oxidation during microscopic observation, we limited the observation time to two minutes. Especially, we could observe photo-induced phase separation in l-menthol containing lipid mixtures, as shown in Figure S4. We ignored such domains that appeared just after light irradiation to avoid any artefacts.

### 2.5. Miscibility Temperature Measurements

We counted 55 liposomes for each composition in each temperature between 18 °C and 40 °C for the systems without Chol, and between 18 °C and 32 °C for the systems with Chol using a thermo-controller, and plotted the fraction of the phase-separated liposomes. We calculated the miscibility temperature (*T*_m_) for the lipid membranes using the obtained experimental plots. The miscibility temperature is defined as the temperature at which the fraction of phase-separated liposome reaches 50%. To obtain the miscibility temperature, the experimental results were fitted with the sigmoidal Boltzmann function

(1) $$p=\frac{1}{1+\exp\left[\left(T-T_{m}\right)/dt\right]}$$

where *p* is the fraction of the phase-separated liposomes, *T* is the temperature, *T*_m_ is the miscibility temperature, and *dt* is the slope of the sigmoidal curve.

## 3. Results 

### 3.1. NMR Spectroscopy

^1^H- and ^13^C-NMR measurements were conducted to investigate the interactions between lipid molecules and d- or l-menthol. As shown in Figure S1 (supplementary information), DOPC, Chol, and menthol molecules each have hydrophobic regions in their chemical structures which may play key roles for these interactions. Further information regarding the interaction between DOPC and d- or l-menthol was obtained mainly using ^13^C-NMR because its wide peak distribution aids analysis.

According to previous reports [37], ^1^H-NMR peaks characteristic of DOPC at 3.42, 1.30, and 0.895 ppm are assigned as choline methyl, methylene, and terminal methyl protons, respectively. ^1^H-NMR spectra of DOPC, Chol, and menthol at 15 °C are shown in Figure S2 (Supplementary Information). The ^1^H-NMR chemical shift values for DOPC, Chol, and menthol are summarized in Table S1 (Supplementary Information). Since there were no significant differences in chemical shifts between d- and l-menthol, we show only the l-menthol results. The ^1^H- and ^13^C-NMR resonance assignments for DOPC, Chol, and menthol were conducted according to the literature [38,39,40]. The ^1^H-NMR spectrum of DOPC dissolved in CDCl_3_ is very close to that of vesicles dissolved in H_2_O/D_2_O. Thus, in this work, we directly compared the resonance assignment for DOPC vesicles reported by Marzorati et al. [41] and Warschawski et al. [37]. The similarity of NMR spectra strongly supports that local conformation of DOPC dissolved in CDCl_3_ is almost identical to that of DOPC vesicles. 

**Figure 1:**
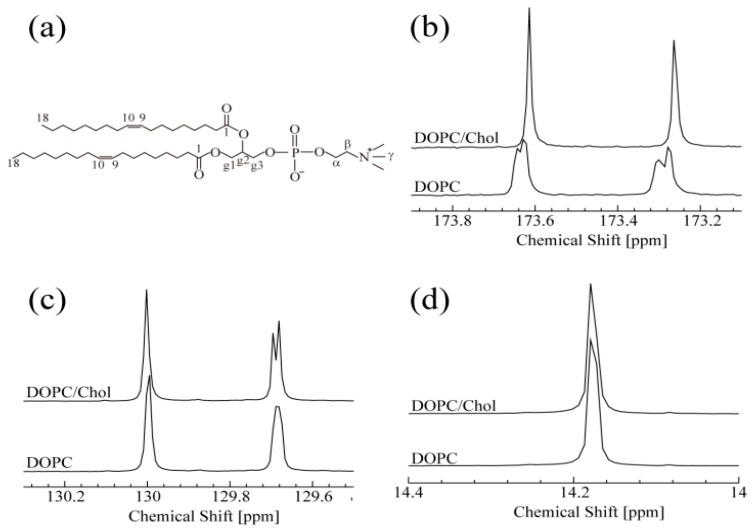
(**a**) Systematic numbering of the chemical structure DOPC. (**b**–**d**) ^13^C-NMR spectra of DOPC and DOPC/Chol systems at 15 °C. (**b**) Both peaks correspond to C1 of DOPC. (**c**) Peaks around 129.7 ppm and 130.0 ppm correspond to C10 and C9 of DOPC, respectively. (**d**) Peaks correspond to C18 of DOPC.

As shown in Figure 1, one-dimensional ^13^C-NMR spectra with ^1^H decoupling were monitored at 15 °C for DOPC with and without Chol. In the absence of Chol, the ^13^C-NMR of DOPC spectrum (Figure 1b) split into peaks, at 173.25 and 173.61 ppm, which correspond to two C1 positions. This splitting may be caused by a certain conformational change around the C1 (carbonyl group) sites. In contrast, in the presence of Chol, each split peak becomes a singlet, suggesting that each C1 site is in a single conformation in the NMR time scale. The observation of two sharp peaks indicates that the two C1 sites in the presence of Chol are in different local magnetic environments, although their line shapes are very similar. Moreover, their chemical shift difference, Δω, appears to remain unchanged by Chol binding. These NMR results strongly suggest that the interaction between Chol and both C1 sites is identical. The two peaks shown in Figure 1c are assigned to C10 (129.7 ppm) and C9 (130.0 ppm) of DOPC. With the addition of Chol, the C10 peak splits, suggesting the existence of two conformations around C10 when DOPC interacts with Chol. Unlike C10, C9 is not affected by Chol. A similar insensitivity was observed for C18, as shown in Figure 1d. These results suggest that C9 and C18 are not directly involved in Chol binding. The DOPC peaks showing spectral changes upon titration with Chol are assigned to the hydrophobic C10 and the hydrophilic C1 site. These findings are consistent with previous results showing that Chol is localized in the hydrophobic core of the bilayer [34]. The interaction between cholesterol and phospholipids is very important in the raft system and it was reported that cholesterol occupies the space between the alkyl chains of lipid tails. Giordani et al. depicted the orientational preference of cholesterol in phospholipid membranes [34]. They have suggested that cholesterol is oriented perpendicular to the bilayer with OH group facing the aqueous pool. Present ^13^C-NMR results on C1 support their report [38]. They also provided evidence of close contact of the hydrophobic portion of cholesterol and hydrophobic tails of phospholipid molecules. In a similar approach, we found the change near both hydrophilic and hydrophobic regions upon addition of cholesterol to the DOPC molecules. Therefore, the hydrophobic part of cholesterol (sterol ring) is localized near C10, and the OH group of cholesterol affects C1. 

**Figure 2:**
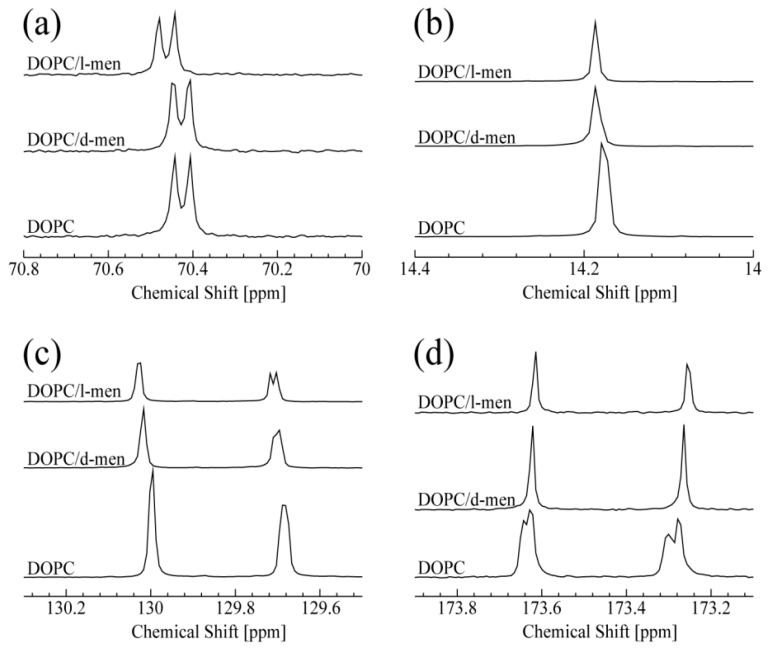
^13^C NMR spectra of DOPC at 15 ^o^C. From the bottom, in the absence and the presence of d- or l-menthol: (**a**) peaks correspond to g2 of DOPC; (**b**) peaks correspond to C18 of DOPC; (**c**) peaks around 129.7 ppm and 130 ppm correspond to C10 and C9 of DOPC, respectively; and (**d**) both peaks correspond to C1 of DOPC.

The obtained ^13^C-NMR spectra of DOPC titrated with d- or l-menthol are summarized in Figure 2. The peak at 70.4 ppm shown in Figure 2a corresponds to the carbon atom at the g2 position of DOPC. It is noteworthy that a clear and significant chemical shift change of the g2 peak occurred only in the presence of l-menthol (also shown in Figure S3 (Supplementary Information)). In Figure S3 (Supplementary Information), a doublet at 70.4 ppm and a singlet at 71.57 ppm are assigned to the g2 position of DOPC and C1 of menthol (see Figure S1b,c (Supplementary Information), respectively). The peak corresponding to C1 of l-menthol shifts to lower field with increasing concentration. Taken together, it could be expected that the hydrophilic interaction exists between g2 of DOPC and C1 of menthol. This could suggest l-menthol can promote hydrophilic interaction at the g2 position. Interestingly, d-menthol does not induce this spectral change for g2 of DOPC. As shown in Figure 2b, both d- and l-menthol slightly shifted the peak at 14.2 ppm, due to resonance from the terminal methyl group at C18 of DOPC. This spectral change indicates that the effect of d- and l-menthol on C18 is likely to be very limited. The peaks at 129.7 and 130.0 ppm, shown in Figure 2c, are assigned to C10 and C9 of DOPC, respectively. Both d- and l-menthol shifted these two peaks down field, although their effect on line shape is different. The difference is caused by their stereochemistry. Since d-menthol does not interact with the hydrophilic part of DOPC (g2 of DOPC), it is thought that d-menthol is preferentially localized to the hydrophobic region of DOPC. Especially, d-menthol is located around the double bond of DOPC, since the peaks for C10 and C9 are affected by d-menthol. As a result, the d-menthol molecules may give a small effect on the peak of C18. Moreover, l-menthol molecules exist in the hydrophilic part of lipids due to the hydrophilic interaction. On the other hand, it is important to note that l-menthol is widely used as a penetration enhancer in clinical studies depending on its concentration [42]. It was reported that, at a very high l-menthol concentration of nearly 30%, l-menthol molecules penetrate into the bilayer (hydrophobic region) significantly, thus leading to perturbation of the lipid tail. Therefore, some l-menthol molecules may exist in the hydrophobic region, in particular, they affect the carbons near the double bond, C10 and C9. As is the case with d-menthol, some l-menthol molecules are located near the double bond of DOPC and affect the peak for C18 consequently.

As shown in Figure 2d, addition of d- or l-menthol causes a similar spectral change in the DOPC C1 peaks at 173.25 and 173.61 ppm, reminiscent of the changes caused by Chol titration, as discussed for Figure 2b. Thus, interaction with d- or l-menthol induces one or two single conformations around the DOPC C1 (C=O) sites. In summary, d- and l-menthol display different specificities in their interaction with DOPC lipid, likely due to their chiral structures allowing them to adopt different conformations. We can predict that both d- and l-menthol exhibits hydrophobic interaction with DOPC. In addition, l-menthol preferentially displays hydrophilic interaction with the hydrophilic part of DOPC. Thus, a chirality-dependent conformational difference is a potential determinant for how d- and l-menthol intrinsically interact with DOPC and provides hints for understanding their different biological effects at atomic resolution. 

### 3.2. Phase-Separated Structure Observation by Microscope

Next, we observed phase separation in lipid membranes containing d- or l-menthol. The concentration of menthol in our experiments of 10% is higher than the concentration for physiological conditions. In this study, however, our main aim is to reveal the interaction of menthol in lipid membranes. Therefore, we performed the experiment at higher menthol concentration to clearly investigate the effects of menthol. In near future, it is necessary to evaluate the effects of menthol on actual living cells with careful attention to the physiological relevance. 

**Figure 3:**
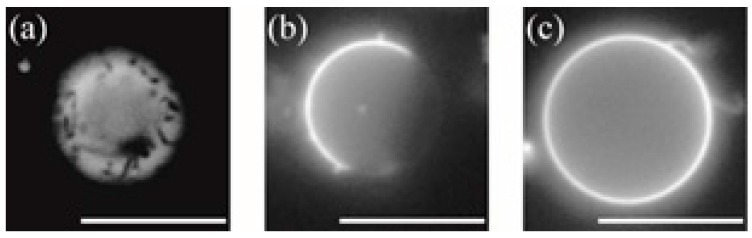
Typical fluorescence microscopic images of S_o_ domain in DOPC/DPPC (50/50) (**a**), L_o_ domain in DOPC/DPPC/Chol (40/40/20) (**b**), and no domain (homogeneous phase) in DOPC/DPPC/Chol (35/35/30) (**c**). Scale bar = 10 μm.

**Figure 4:**
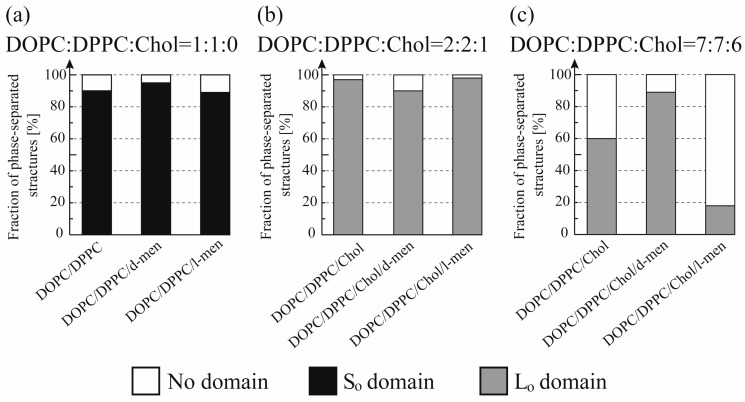
Fraction of phase-separated structures in DOPC/DPPC/Chol/menthol systems at room temperature. We fixed DOPC:DPPC:Chol = 1:1:0 in (**a**), DOPC:DPPC:Chol = 2:2:1 in (**b**), and DOPC:DPPC:Chol = 7:7:6 in (**c**). The mole fraction of menthol is 10%. White, black, and grey bars indicate no domain (homogeneous phase), S_o_ domain formation, and L_o_ domain formation, respectively. Number of liposomes counted = 55.

Figure 3 and Figure 4 show typical fluorescence microscopic images and the fraction of different types of phase-separated membranes. First, we observed the phase separation of a binary DOPC/DPPC and ternary DOPC/DPPC/Chol mixture at room temperature. In a binary system without Chol, DPPC-rich stripe like (S_o_) domains (dark region) formed predominantly surrounded by DOPC-rich L_d_ phase (bright region) as shown in Figure 3a. At room temperature we could not find a clear difference of the phase behaviour by adding d-/l-menthol, as shown in Figure 4a. Next, we observed the phase separation in DOPC/DPPC/Chol with menthol. The fluorescent dye Rho-DHPE is localized in the DOPC-rich L_d_ phase; circular shapes (dark region) L_o_ domains enriched with DPPC and Chol surrounded by L_d_ phase were formed in ternary system DOPC/DPPC/Chol = 40/40/20 in Figure 3b. Additionally, there is no significant difference between the system without menthol and d-/l-menthol containing systems as shown in Figure 4b. At Chol = 30% system, we found significant effects by d- and l-menthol on phase behaviour in Figure 4c. The phase-separated structure is stabilized by d-menthol, whereas l-menthol significantly suppresses the phase separation. 

**Figure 5:**
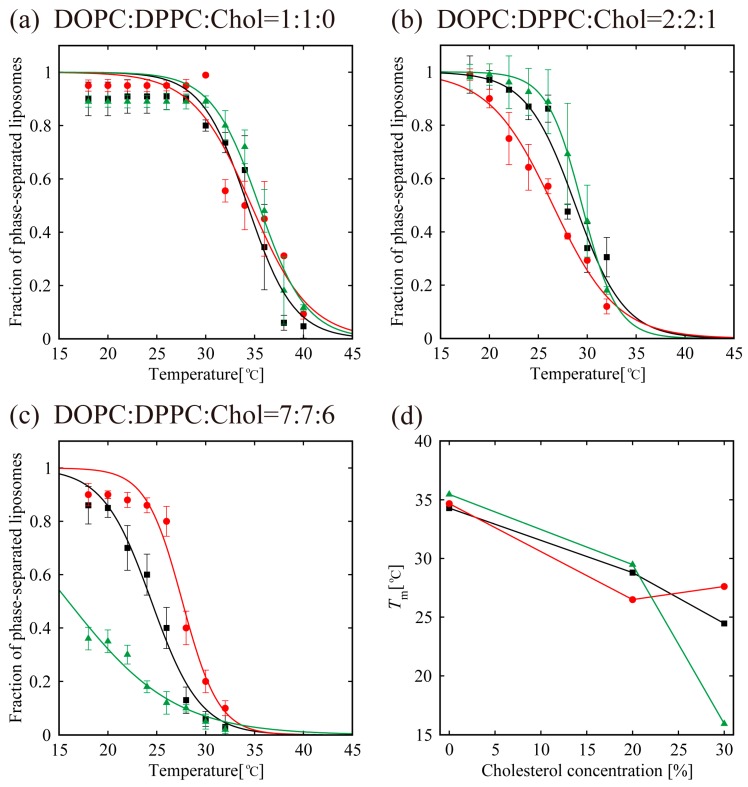
Miscibility temperature measurement for DOPC/DPPC/Chol/menthol systems. We fixed DOPC:DPPC:Chol = 1:1:0 in (**a**), DOPC:DPPC:Chol = 2:2:1 in (**b**), and DOPC:DPPC:Chol = 7:7:6 in (**c**). The mole fraction of menthol is 10%. Black lines and black squares indicate DOPC/DPPC/Chol without menthol. Red lines with red circles and green lines with green triangles are DOPC/DPPC/Chol with d-menthol and l-menthol, respectively. (**d**) The change in the miscibility temperature as a function of Chol concentration in lipids, defined as [Chol]/([DOPC]+[DPPC]+[Chol])%.

In order to understand the effects of d- or l-menthol on the phase separation more clearly, the miscibility temperature was observed over the temperature range from 18 °C to 38 °C using a thermo-controller. First, we observed the phase separation of the binary lipid mixtures consisting of the unsaturated lipid DOPC and the saturated lipid DPPC without Chol at a temperature ranging from 18 °C to 38 °C (obtained using the thermo controller). To examine the miscibility temperature, we measured the fraction of phase-separated structures over 55 liposomes in each system. The miscibility temperature *T*_m_ is defined as the temperature at which 50% of GUVs become heterogeneous, as mentioned in the Materials and Methods. Some studies reported the concentration fluctuated slightly above the critical temperature [43,44]. In our experiment, the temperature above the miscibility temperature is about *T* = 40 °C, which is close to the physiological temperature. Although this temperature range is important, biologically, we could not observe such an interesting phenomenon. Therefore, we focus only on the miscibility temperature. Figure 5a shows the fraction of DOPC/DPPC liposomes forming phase-separated structures at each temperature tested. Black-filled squares and lines show the results obtained in the absence of menthol, and the red and green symbols and lines correspond to d- or l-menthol containing liposomes, respectively. The symbols (squares, circles, and triangles) denote experimental data and lines were obtained from Equation (1) to fit the experimental results. The phase separation in the DOPC/DPPC (Chol = 0%) was observed. No clear difference in the miscibility temperature was obtained by adding d- or l-menthol to this binary system, as shown in Figure 5a,d, nor was there a clear difference in phase-separated structures at room temperature upon addition of d- or l-menthol (Figure 4a).

Next, we observed phase separation in the DOPC/DPPC/Chol (Chol = 20%) ternary lipid mixture. Circularly-shaped L_o_ domains enriched with DPPC and Chol (dark regions) surrounded by the L_d_ phase (bright region), as shown in Figure 3b, were mainly formed in the DOPC/DPPC/Chol = 40/40/20 ternary system. Additionally, the system without menthol and the d-/l-menthol-containing systems showed no significant differences in miscibility temperature, as shown in Figure 5b,d, or in phase-separated structures at room temperature (Figure 4b). At Chol = 30%, however, clear and significant phase behaviour differences induced by d- or l-menthol were apparent, as shown in Figure 5c,d. Notably, d-menthol stabilized phase separation, whereas l-menthol dramatically lowered the phase-separated fraction. The same tendency is also seen in Figure 4c. Figure 5d shows the change in the miscibility temperature as a function of Chol concentration: the miscibility temperature clearly tends to be higher for d-menthol and is lower for l-menthol at Chol = 30%. During microscopic observation, the transformation from homogeneous to L_d_ domains surrounded by the L_o_ phase (reverse domains) in l-menthol-containing membranes at Chol = 30% occurs just after excitation with light irradiation, as shown in Figure S4 (Supplementary Information). This result implies that the homogeneous phase may be a metastable phase. However, we ignored this transformation, since artefacts, including lipid oxidation by light irradiation, should first be eliminated. 

## 4. Discussion

In the absence of Chol (Chol = 0%), the addition of d-/l-menthol does not affect the phase behaviour as shown in Figure 4a and Figure 5a. NMR measurements revealed the probable interaction sites between d-/l-menthol and lipids. It can be concluded that d-menthol affected the hydrophobic region, whereas l-menthol incurred disordering effects to both hydrophilic and hydrophobic regions. We assume d-menthol is localized in the DOPC-rich L_d_ phase because d-menthol cannot be accommodated in the tightly packed DPPC-rich S_o_ phase. Therefore, d-menthol does not affect S_o_ domain formation. On the other hand, l-menthol interacts with the hydrophilic region (phosphocholine: PC) and, thus, might interact with DOPC and DPPC in the same manner. Since the number density of lipid molecules in the S_o_ phase is larger than that in the L_d_ phase, l-menthol preferentially localizes in the S_o_ phase. As a result, the hydrophilic region of the S_o_ phase becomes crowded and steric repulsion may arise. However, the attraction between DPPC molecules is sufficiently strong that the addition of l-menthol does not disturb the domain formation in DOPC/DPPC mixtures. This tendency is similar to the case of the Chol = 20% DOPC/DPPC/Chol mixture demonstrated in Figure 4b and Figure 5b. From Figure 4c and Figure 5c, at Chol = 30%, however, d-menthol stabilizes L_o_ domain formation. Here, we consider that there is an attraction between d-menthol and Chol as d-menthol is localized around the hydrophobic core similarly to Chol. As mentioned above, d-menthol may partition preferentially into the L_d_ phase. Some Chol molecules in the L_o_ phase migrate to the L_d_ phase due to the attraction between d-menthol and Chol. As a result, the amount of Chol in the L_o_ phase is decreased, stabilizing L_o_ domains, because the homogeneous phase exhibits at higher Chol concentration (e.g., DOPC/DPPC/Chol (30/30/40)) [19]. Because both d-menthol and Chol exist in hydrophobic region, the direct attraction between d-menthol and Chol may be present. Since the direct attraction between d-menthol and Chol is our speculation, it is important to investigate the interaction by some additional experiments in the future. In contrast, phase separation was strongly suppressed by the addition of l-menthol to DOPC/DPPC/Chol at Chol = 30% (see Figure 5c). As stated above, l-menthol may be localized in the densely packed DPPC-rich L_o_ phase due to the high Chol concentration (Chol = 30%), lipid packing in the L_o_ phase is weaker compared with the case of Chol = 20%. In addition, l-menthol may partition largely into the L_o_ phase, which includes large amounts of Chol due to the cooperative interactions among PC, the hydroxyl group of Chol, and l-menthol. Therefore, steric repulsion by l-menthol in the crowded hydrophilic region significantly disturbs L_o_ domain formation. 

In the present NMR study, the system was prepared in chloroform. It was predicted that the interactions in chloroform would be different from those in the lipid bilayer. However, we believe that the interpretations in hydrophobic parts are reasonable, because the carbons present in hydrocarbon tails are of a hydrophobic nature even in the bilayer or chloroform. However, the carbons in the hydrophilic part become an important concern as in the bilayer they are in a hydrophilic environment, whereas they are in a hydrophobic environment in chloroform. Since our NMR study was performed in a CDCl_3_ environment, that is, a hydrophobic environment, the hydrophilic interaction might become stronger than that in a hydrophilic environment. Even in a hydrophobic environment, however, d-menthol does not clearly interact with the hydrophilic part of lipids. Therefore, we can imagine that the hydrophilic interaction between d-menthol and the hydrophilic head group are also ignored in a hydrophilic environment. On the other hand, l-menthol may potentially interact with the hydrophilic part in a hydrophilic environment. Moreover, the solubility of menthol in water is known to be very low. This implies that menthol does not interact with water molecules favourably. Hence, it is reasonable that the hydrophilic interaction between l-menthol and the hydrophilic part of lipids is predominant even in a bilayer nature. Therefore, we considered that these observations were further supported by the GUV experiment. To confirm the interaction between menthol and lipids in a bilayer nature, we will need to perform additional experiments, such as using NOESY on 800 MHz-NMR. 

These findings can be explained by considering the change in the lipid bilayer physical state near the phase transition. The phase transition is referred to as the transition between the gel state, i.e., solid-ordered phase, to fluid, i.e., the liquid-disordered phase. From the results obtained we proposed a model showing the possible interaction of d-/l-menthol with a lipid bilayer which could affect the cold-sensing properties of TRPM8. We also believe that TRP channels can sense the temperature-dependant changes in the lipid bilayer. Briefly, a rise in the temperature and cholesterol concentration could alter the lipid packing, indeed leading to changes in the membrane protein function, such as that involved in the function of the channel. It is interesting to speculate that since l-menthol has shown hydrophilic interaction predicted by ^13^C-NMR at g2 and C1(C=O) positions of the lipid molecule, this interaction could be influenced by temperature. Previous studies [45] support our data obtained from ^13^C-NMR regarding the hydrogen bond between the ester group of PC (C=O) and the hydroxyl group of Chol. At lower temperature, Chol movement is restricted in the bilayer, thereby stabilizing the membrane structure that corresponds to cold sensitization at cold temperatures. The phase behaviour data was demonstrated at lower Chol concentration, and l-menthol displays strong hydrophilic interaction stabilizing the head group interaction, which could relate to cooling phenomena even at low temperature. On increasing temperature, as well as cholesterol concentration, the same interaction now becomes more hindered and mobile to cause head group expansion, thus destabilizing the lipid structure. On the other hand, d-menthol exhibits hydrophobic interaction localized in the hydrophobic region with Chol causing strong hindrance and, disturbing the packing, i.e*.,* destabilizing the membrane. It was reported that some of the TRP channels are sensitive to the cholesterol content and is important in channel functioning [46]. We strongly believed that the hydrophilic interaction around the g2 and C1 (C=O) positions of the lipid molecule plays an important role in modulation of TRPM8 channel activity. Some amino acid residue of the TRPM8 channel may be involved in those temperature sensitive interactions at hydrophilic surface incriminated in the cold sense by l-menthol. Furthermore, these dynamic changes in the membrane properties enable picturing the related mechanism behind the cooling sensation. These experiments persuade further attention to the regulation of the membrane protein channel activity by the lipid environment. 

## 5. Conclusions

In this paper, we investigated the chirality-dependent interaction of the two optical isomers menthol (d- and l-) on the stability of membrane heterogeneity. Phase behaviour of the DOPC/DPPC/Chol system at different Chol concentrations was studied thoroughly in the presence of d- or l-menthol. Miscibility temperature measurements demonstrated the importance of the interaction between menthol and cholesterol in biological processes. Our findings provide strong evidence of the core importance of Chol in biomembranes. Furthermore, we predicted the induction of different regions of the biomembrane by d- and l-menthol, and this induction strongly depends on the Chol concentration. These findings may aid future understand of the cooling phenomenon caused by menthol and cold compounds.

## Supplementary Materials

The following are available online at www.mdpi.com/2077-0375/7/04/69/s1, Figure S1: Systematic numbering of the chemical structures of (a) Chol, (b) l-menthol, and (c) d-menthol.; Figure S2. ^1^H-NMR spectra of (a) DOPC, (b) Chol, and (c) menthol.; Figure S3. ^13^C-NMR spectra of DOPC titrated with d- or l-menthol at 15 °C; Figure S4: Typical microscopic image of reverse domain formation. Scale bar = 10 μm. Table S1: 1H chemical shift values for DOPC, Chol, and menthol at 15 °C. Since there are no significant differences in chemical shifts between d- and l-menthol, only the results for l-menthol are shown.
