# Supplementary material for: Chirality-Dependent Interaction of d- and l-Menthol with Biomembrane Models
Source: Membranes (Basel). 2017 Dec 15;7(4):69. doi: 10.3390/membranes7040069 (PMC5746828; doi:10.3390/membranes7040069)
Supplement: Supplementary file 1 [file membranes-07-00069-s001.pdf]

Electronic Supplementary Information

Chirality-Dependent Interaction of d- and l-Menthol with Biomembrane Models

Pooja Gusain [1], Shinya Ohki [1], Kunihide Hoshino [1,2], Yoshio Tsujino [1,2], Naofumi Shimokawa [1], and Masahiro Takagi *[1]

[1] *School of Materials Science, Japan Advanced Institute of Science and Technology, 1-1 Asahidai, Nomi, Ishikawa 923-1292, Japan.*

[2] *Takasago International Corporation, 5-37-1, Kamata, Ota-ku, Tokyo 144-8721, Japan.*

NMR Spectroscopy

The ^{13}C-NMR spectrum of DOPC titrated with d- or l-menthol at 15 °C is shown in Figure S3. As the concentration of d-menthol increased, the peak at 70.4 ppm, corresponding to the carbon at the g2 position of DOPC, remained unchanged. On the other hand, this peak became less intense as the l-menthol concentration was increased. In addition, the peak at 71.57 ppm, which corresponds to C1 of menthol, intensified compared to when d-menthol was added. Since the carbon at the g2 position is located in the hydrophilic region of DOPC, there is a hydrophilic interaction between l-menthol and DOPC.

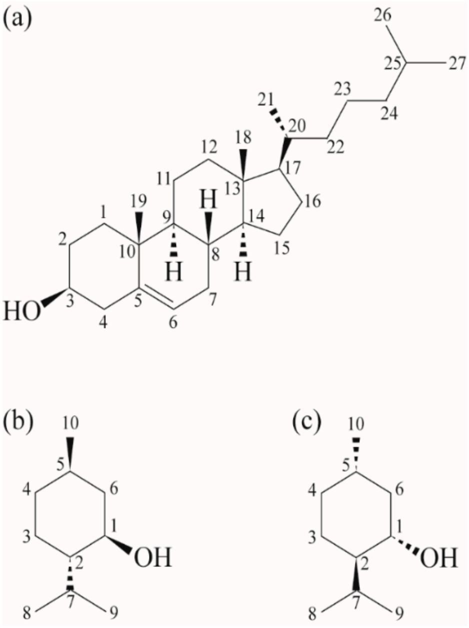

Figure S1. Systematic numbering of the chemical structures of (**a**) Chol, (**b**) l-menthol, and (**c**) d-menthol.

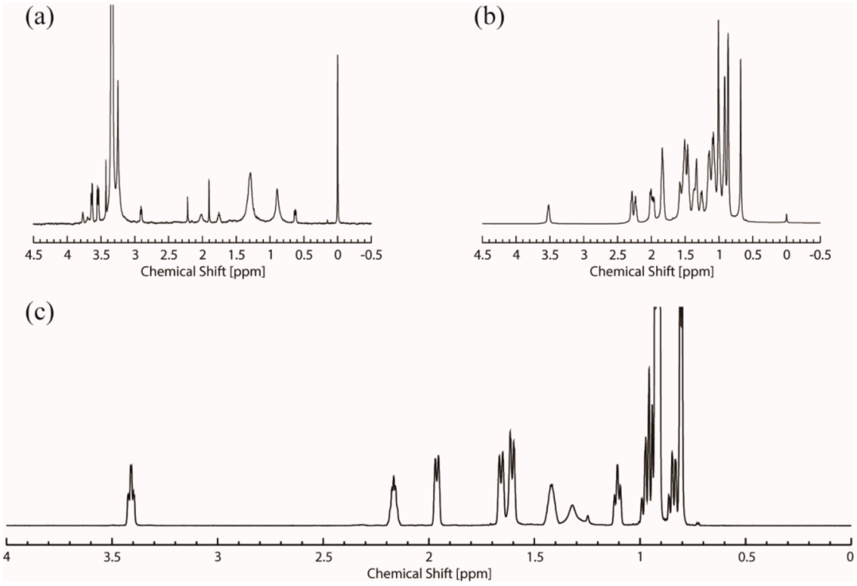

Figure S2. ^{1}H-NMR spectra of (**a**) DOPC, (**b**) Chol, and (**c**) menthol.

Component	^{1}H-site	Chemical Shift (ppm)
**DOPC**	C18	0.895
	α	3.42
	C9 and C10	1.90
	$(CH_2)_n$	1.30
	C1	2.01
**Chol**	C26-27	0.88
	C25	1.56
**Menthol**	C1	3.41
	C6	1.96
	C7	2.16
	C8	0.93
	C9	0.809
	C10	0.90
	C5	1.41

Table S1. ^{1}H chemical shift values for DOPC, Chol, and menthol at 15 °C. Since there are no significant differences in chemical shifts between d- and l-menthol, only the results for l-menthol are shown.

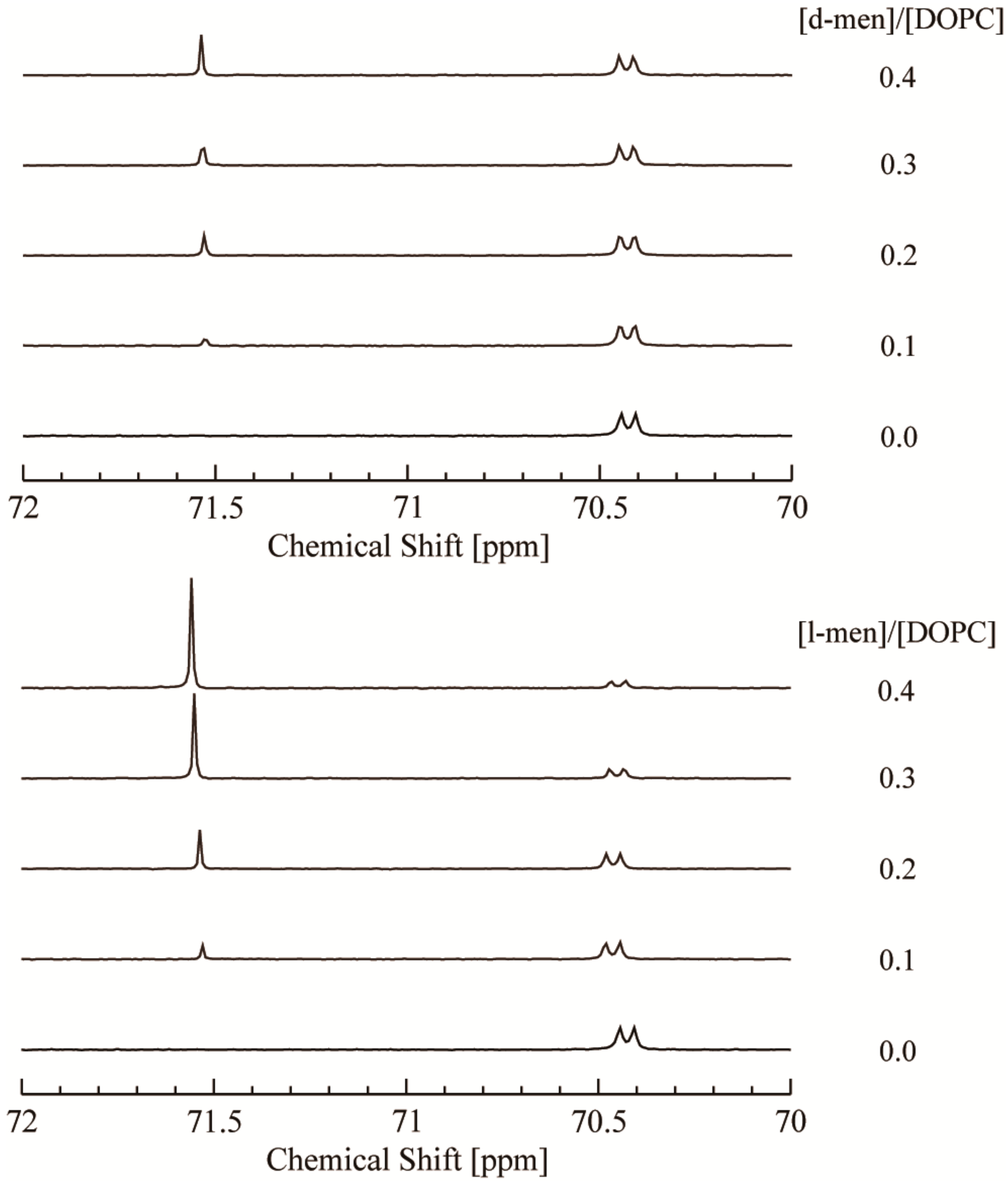

Figure S3. ^{13}C-NMR spectra of DOPC titrated with d- or l-menthol at 15 °C.

Phase-separated Structures at Room Temperature

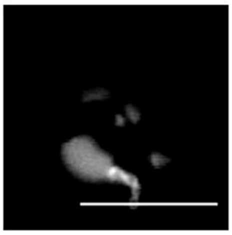

Figure S4. Typical microscopic image of reverse domain formation. Scale bar = 10 μm.
